# PEG-Immobilized Keratin for Protein Drug Sequestration and pH-Mediated Delivery

**DOI:** 10.1155/2016/7843951

**Published:** 2016-01-20

**Authors:** Roche C. de Guzman, Sina Y. Rabbany

**Affiliations:** Bioengineering Program, Department of Engineering, Hofstra University, Hempstead, NY 11549, USA

## Abstract

Protein drugs like growth factors are promising therapeutics for damaged-tissue repair. Their local delivery often requires biomaterial carriers for achieving the therapeutic dose range while extending efficacy. In this study, polyethylene glycol (PEG) and keratin were crosslinked and used as sponge-like scaffolds (KTN-PEG) to absorb test proteins with different isoelectric points (pI): albumin (~5), hemoglobin (~7), and lysozyme (~11). The protein release kinetics was influenced by charge at physiological pH 7.4. The keratin network, with pI 5.3, electrostatically attracted lysozyme and repulsed albumin generating the release rate profile: albumin > hemoglobin > lysozyme. However, under acidic conditions (pH 4), all proteins including keratins were positively charged and consequently intermolecular repulsion altered the release hierarchy, now determined by size (MW) diffusion: lysozyme (14 kDa) > hemoglobin (64 kDa) > albumin (66 kDa). Vascular endothelial growth factor C (VEGF-C), with properties comparable to lysozyme, was absorbed into the KTN-PEG scaffold. Endothelial cells cultured on this substrate had significantly larger numbers than on scaffolds without VEGF-C suggesting that the ionically bound and retained growth factor at neutral pH indirectly increased acute cell attachment and viability. PEG and keratin based sequestrations of proteins with basic pIs are therefore a feasible strategy with potential applications for selective biologics delivery.

## 1. Introduction

Protein drugs, also called protein therapeutics and protein biologics, are proteins that provide healing, repair, and regenerative functionalities to injured and damaged cells and tissues. These include cell-secreted extracellular growth factors (GFs) and signaling proteins for induction of cell growth, cell division (proliferation), movement, changes in shape, survival and inhibition of apoptosis, differentiation, and tissue morphogenesis [1–4]. GFs bind to target enzyme-linked cell-surface receptors and activate intracellular signaling pathways leading to expression of genes involved in macromolecular synthesis, metabolism, and alteration of cellular behaviors. Effective levels of GFs are usually in the pico- to nanomolar ranges, acting in the order of hours [5]. To extend this range and regulate the spatiotemporal dose release for treatment applications, drug-delivery systems or biomaterial carriers are utilized [6–8]. A charge-based sequestration strategy can be employed wherein the protein drug load and the carrier matrix have opposing electrical charges to provide electrostatic or coulombic attraction. Proteins and peptides are zwitterions; that is, they can alter their net charges depending on the aqueous environmental pH; those with isoelectric points (pIs) lower than the pH of the medium will have negative molecular electrical charges, while those with higher pIs will be positively charged [9, 10]. Accordingly, proteins with acidic and basic pIs in phosphate-buffered saline (PBS, pH 7.4) carry negative and positive net charges, respectively, and can potentially associate together.

**Figure 1:**
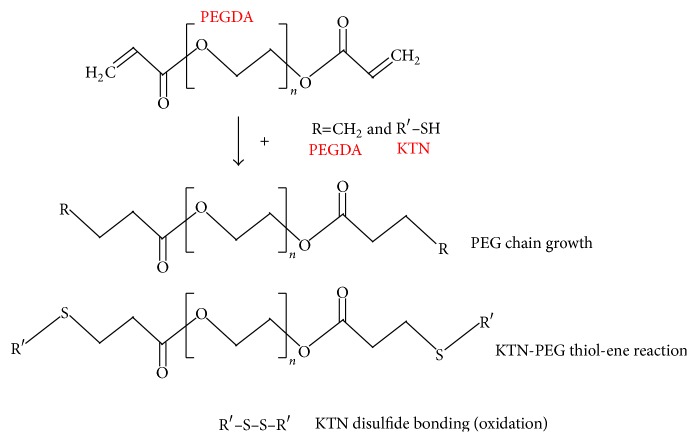
Crosslinking reactions of polyethylene glycol diacrylate (PEGDA) and keratin (KTN) mixture.

Polyethylene glycol (PEG) and hair keratin (KTN) biomaterials have been shown to be safe, biocompatible (with minimal fibrous encapsulation), and appropriate drug-delivery vehicles for tissue engineering purposes [11–19]. Diacrylates of linear PEG (PEGDA) can be photopolymerized for PEG chain growth to form scaffolds [20, 21]. Reduced KTN proteins containing free thiols (–SH) can be gelled by reforming of disulfide bonds (–S–S–) [22, 23]. PEGDA and KTN can also be combined and crosslinked via a photopolymerization thiol-ene reaction [24, 25]. We capitalized on these reactions (Figure 1) to form stable KTN-PEG scaffolds. KTN, having a pI of 5.3, is negatively charged in PBS, pH 7.4 (Table 1) [22]. Moreover, the reactions do not target any of the ionizable amino acid groups [9] and should not significantly alter the crosslinked keratin pI. The KTN network of KTN-PEG, hence, can theoretically hold onto positively charged proteins with basic pIs. Several GFs, including vascular endothelial growth factor C (VEGF-C), exhibit pIs greater than 7.4 [26, 27]. As a result, they are likely to ionically associate with the KTN-PEG bulk material. In this study, we investigated the diffusion release profiles of KTN-PEG scaffold-absorbed soluble proteins with varying pIs (charges) and sizes in physiological pH and, additionally, in acidic PBS, pH 4 (Table 1). At a pH level lower than the KTN's pI, the biomaterial scaffold is expected to gain positive charges, thereby inducing repulsion and quick release of sequestered positively charged proteins. The functional bioactivity of a bound-protein, represented by VEGF-C, was tested through endothelial cell culture onto the KTN-PEG substrate with absorbed VEGF-C.

## 2. Materials and Methods

### 2.1. Materials

The following reagents and proteins were purchased from Sigma-Aldrich (St. Louis, MO): polyethylene glycol diacrylate (PEGDA; *M*_*n*_ = 700 g/mol; *n* ~ 13), glycerol, methanol (MeOH), NaOH, HCl, phosphate-buffered saline (PBS; pH = 7.4), Na_2_CO_3_, NaHCO_3_, Tween 20 (Polysorbate 20), 3,3′,5,5′-tetramethylbenzidine (TMB substrate), calcein AM, propidium iodide, bovine serum albumin (BSA; pI ~ 5, MW = 66 kDa), human hemoglobin (pI ~ 7, MW = 64 kDa), and chicken egg white lysozyme (pI ~ 11, MW = 14 kDa) [28]. Antibodies, goat anti-BSA (sc-50710), goat anti-human hemoglobin (sc-31110), goat anti-hen egg lysozyme (sc-325025), and anti-goat IgG-HRP (sc-2020), were bought from Santa Cruz Biotechnology (Dallas, TX). The test growth factor, human vascular endothelial growth factor C (VEGF-C), was obtained from ACROBiosystems (Newark, DE). Acidic PBS (pH = 4) was prepared by dropwise addition of HCl. Irgacure^®^ 2959 (I2959), provided by BASF (Ludwigshafen, Germany), was dissolved at a stock solution of 10% (m/V) in MeOH. Reduced keratin biomaterial (KTN; MW = 98 kDa) was extracted [22] and obtained from Mark Van Dyke's Lab at the School of Biomedical Engineering and Sciences, Virginia Tech (Blacksburg, VA). Distilled deionized water (at 18.2 MΩ·cm electrical resistivity) was used as the default aqueous solvent, unless otherwise indicated.

### 2.2. Scaffold Fabrication

**Figure 2:**
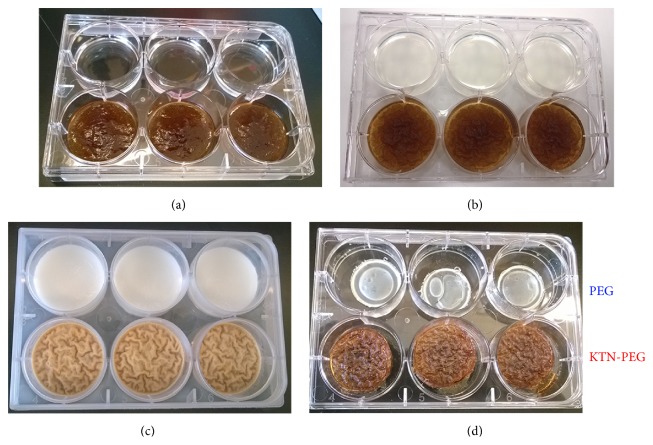
Preparation of scaffolds: (a) keratin gelation via disulfide bond formation of intermolecular chains, (b) photopolymerization reaction to increase polyethylene glycol molecular weight and to crosslink to keratin, (c) freezing, and (d) lyophilization to remove unbound water, finally producing PEG and KTN-PEG constructs.

KTN-PEG scaffolds were prepared for prereaction concentration of 20% (V/V) PEGDA, 10% (V/V) glycerol, 1% I2959 (m/V), and 5% (m/V) KTN in 10 mM NaOH solution at 3 mL per well in 6-well plates. The mixture was thoroughly stirred until being homogenous while minimizing air bubbles. Using DC Protein Assay (Bio-Rad, Hercules, CA), the initial keratin concentration was verified to be 48.7 ± 9.8 mg/mL, within the expected 50 mg/mL (or 5%) KTN. Disulfide bond formation (via oxidation of thiols and direct thiol-thiol interaction) of keratin chains was ensured by overnight incubation at 4°C (Figure 2(a)) [22, 23]. Polyethylene glycol (PEG) network formation (PEGDA-PEGDA; PEG chain growth) and thiol-ene reaction for crosslinking keratins to PEG were made via exposure to UV light at 254 nm for 20 min (Figure 2(b)). The energy to polymerize was computed to be (9 W/bulb) · (4 bulbs) · (1200 s) = 43200 J = 43.2 kJ. After freezing at −80°C (Figure 2(c)), samples were then lyophilized using FreeZone 2.5 Plus (Labconco, Kansas City, MO) for 24 hr (Figure 2(d)). Control PEG constructs were fabricated similarly except for the absence of KTN. Excess glycerol plasticizer [29] that did not integrate into the network was blotted out.

### 2.3. Liquid Absorption and Stability

PEG and KTN-PEG scaffolds were tested for their ability to absorb PBS. Samples were immersed in PBS and weighed at time points: 10 and 30 min and 1, 2, 14, 24, 48, and 72 hr. The ratio of absorbed liquid (absorbate) to scaffold mass (*m*_absorbate_/*m*_scaffold_) was plotted as a function of time. Caliper measurements of radius = *r* and thickness = *h* of scaffold discs were employed to approximate the bulk cylindrical volume, *V* = *πr* ^2^ *h*.

### 2.4. Crosslinked Network Properties

The following properties of PEG and KTN-PEG scaffolds were quantified [23, 30–32]: *Q*, *ν*_*e*_, and *M*_*c*_. Consider

(1) $$\begin{array}{c} Q=\frac{V_{swollen}}{V_{dry}}, \end{array}$$

where *Q* is equilibrium volume swelling ratio, *V*_swollen_ is volume of absorbate and scaffold, and *V*_dry_ is volume of scaffold. Consider

(2) $$\begin{array}{c} \nu_{e}=\frac{E_{c}}{{RTQ}^{1/3}}, \end{array}$$

where *ν*_e_ is effective crosslink density (mol/mL), *E*_*c*_ is compressive modulus of elasticity (MPa), *R* is gas constant = 8.314 mL·MPa·K^−1^·mol^−1^, and *T* is temperature (K). *E*_*c*_ values were obtained from the slope of the linear elastic region of true stress versus true stain curve of equilibrated PBS-soaked scaffolds compressed at 0.1 mm/s using the Instron 3345 mechanical tester (Norwood, MA). Consider

(3) $$\begin{array}{c} M_{c}=\frac{\rho_{polymer}}{\nu_{e}}, \end{array}$$

where *M*_*c*_ is molecular weight between crosslinks (g/mol) and *ρ*_polymer_ is polymer density (g/mL).

### 2.5. Release of Uncrosslinked Network Proteins

Representative KTN-PEG discs were cut into smaller pieces of known volume, placed into 1.5 mL flip-top tubes with 1 mL PBS, and incubated at 37°C. Aliquots of the PBS medium were collected at 2, 4, 14, and 24 hr and 2, 4, and 7 d. At each time point, fresh PBS was added at equal volume to replenish the sampled liquid. DC Protein Assay was used to quantify the KTN concentration ([KTN]) of the collected samples.

### 2.6. Loading and Release of Test Proteins

KTN-PEG scaffolds were extensively washed to remove uncrosslinked materials including “free” keratins. Samples were immersed in PBS, incubated with shaking for 2 hours, and spent PBS removed and replaced with fresh PBS. Washing was performed trice and the final wash subjected to overnight incubation. Model globular proteins, albumin (Alb), hemoglobin (Hem), and lysozyme (Lys), were diluted with PBS to a stock concentration of 2 mg/mL. Approximately 0.3 cm^3^ (=0.3 mL) scaffolds were individually placed in microfuge tubes and 1 mL of 200 *μ*g/mL Alb, Hem, or Lys in PBS was added. Proteins were allowed to be absorbed into the gel matrix overnight at 4°C. The following day, excess protein solutions were removed by aspiration and scaffolds were quickly rinsed with either PBS or acidic PBS, ×3 at 1 mL each. Gels were left in 1 mL of either PBS or acidic PBS. Protein release samples were collected at time points: 2, 6, and 24 hr and 2, 4, and 6 d. Fresh medium at equal volume was added after each collection. Total protein concentrations were measured through the DC Protein Assay, while specific protein release profiles were assessed using enzyme-linked immunosorbent assay (ELISA).

### 2.7. ELISA Detection

Experimental samples and protein standards were neutralized (for acidic samples) and diluted in carbonate coating buffer (3.03 g Na_2_CO_3_ and 6 g NaHCO_3_ per 1 L; pH 9.6), placed in NUNC MaxiSorp^TM^ (Fisher Scientific, Hampton, NH) 96-well plates at 100 *μ*L per well, and incubated overnight at 4°C. After washing with PBS twice, wells were blocked (300 *μ*L) with blocking buffer (1% (m/V) nonfat dry milk in PBS) for 2.5 hr and then washed twice with PBS-T (0.05% (V/V) Tween 20 in PBS). Samples were hybridized with primary antibody (100 *μ*L; anti-BSA at 1 : 100, anti-human hemoglobin at 1 : 200, or anti-hen egg lysozyme at 1 : 200 in blocking buffer) for 2 hr, washed ×4 with PBS-T, hybridized again with secondary antibody conjugate (100 *μ*L; anti-goat IgG-HRP at 1 : 1000 for Alb or 1 : 5000 for Hem and Lys in blocking buffer) for 1 hr, and then washed ×4 with PBS-T. Color was developed by adding 100 *μ*L of TMB substrate and the reaction was stopped after 15 min with 0.5 N HCl (100 *μ*L). Light absorbance at 450 nm was read using iMark^TM^ (Bio-Rad) spectrophotometer.

### 2.8. Bioactivity of Sequestered Growth Factor

KTN-PEG scaffolds were fabricated on a 24-well tissue culture plate (600 *μ*L per well) and then washed with PBS overnight. VEGF-C (1 mL at 2.5 *μ*g/mL in PBS) was absorbed overnight and scaffolds subsequently were washed with PBS followed by culture medium (CM) composed of Dulbecco's Modified Eagle Medium (DMEM), 20% (V/V) fetal bovine serum, and antibiotics. Control groups without keratin and/or VEGF-C were also included. Human umbilical artery endothelial cells (HUAECs; Sigma-Aldrich) in CM were seeded at 10^4^ cells per well and cultured at 37°C, 5% CO_2_, and 90% relative humidity. CM was removed and replenished after 2 days. At day 5, CM was aspirated out, scaffolds were rinsed with PBS and overlaid with 4 *μ*M calcein AM and 5 *μ*M propidium iodide in PBS for 10 min. After rinsing in PBS, cells were imaged and counted using an inverted fluorescence microscope at both green and red filters (for live and dead cells, resp.) under a ×4 objective field-of-view.

### 2.9. Sample Management and Statistical Analyses

Experimental replicates were conducted with sample sizes of *n* ≥ 3. Computed values, plots, and bar graphs were reported as average ±1 standard deviation. Charts including scatterplots and trendlines were generated using MATLAB (MathWorks, Natick, MA) scripts with LaTeX symbolics. Excel Solver (Microsoft, Redmond, WA) and Prism (GraphPad, San Diego, CA) were employed for the nonlinear curve fitting to coefficient of determination (*r* ^2^) approaching 1. Student's *t*-test and one-way analysis of variance (ANOVA) combined with Tukey's post hoc multiple comparison analyses were done with Prism at 95% confidence intervals and 5% probability of type I error (*α*).

## 3. Results and Discussion

### 3.1. Equilibrium Swelling and Crosslinking Properties

**Figure 3:**
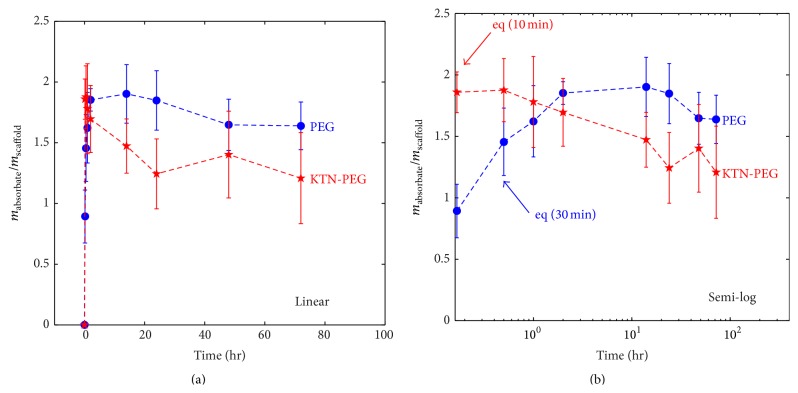
Swelling behavior of PEG and KTN-PEG scaffolds with time at linear (a) and semilog (b) scales. Equilibrium (eq) was attained at 30 min for PEG and at 10 min for KTN-PEG. KTN-PEG samples absorbed water faster due to network-associated hydrophilic keratin proteins.

Control PEG scaffolds (without keratins) reached liquid absorption equilibrium at half an hour in PBS (Figure 3: PEG). ANOVA and Tukey's test showed no significant differences (*p* > 0.05) among groups from the 30 min time point and later. KTN-PEG, on the other hand, had an almost instantaneous equilibration time (Figure 3: KTN-PEG). All of the experimental time groups for KTN-PEG generated statistically similar (*p* = 0.0625) liquid absorbate-to-scaffold mass ratio. At the 10 min time point, KTN-PEG yielded a higher ratio (*p* = 0.0036) compared to PEG (1.86 ± 0.17 versus 0.89 ± 0.22, resp.), indicating that the presence of keratin led to a twofold (1.86/0.89 = 2.08) mass increase in hydrophilicity of the material. Keratins bound to the PEG matrix therefore facilitated more water absorption. The volumetric increase from lyophilized to PBS-wetted KTN-PEG scaffolds after 24 hours was found to be 304 ± 1% or a 3-fold size increase. From the initial prereaction mix volume of 3 mL (Figure 2(a): KTN-PEG), the soaked scaffold swelled to 3.99 mL or a volumetric ratio increase of 133 ± 2%. Conservation of mass principles enabled the computation of several KTN-PEG properties displayed in Table 2.

Table 3 summarizes the results obtained from measurement of scaffold network properties. It was found that the molecular weight between crosslinks (*M*_*c*_) in KTN-PEG scaffolds immersed in PBS was 25.9 kDa. A 5% KTN gel only was determined to have *M*_*c*_ = 183 kDa. Since *M*_*c*_ was reduced in the presence of PEG additive, this suggests successful photocrosslinking of keratin-to-PEG via thiol-ene reaction. The average molecular weight (MW) of the KTN biomaterial was reported to be 98 kDa [22]. Accordingly, there were about 3.8 (=98 kDa/25.9 kDa) crosslinks per KTN molecule in the fabricated KTN-PEG scaffold. In the KTN-PEG scaffold, any of the possible reactions illustrated in Figure 1 can occur: PEG-PEG chain growth, KTN-KTN disulfide oxidation, and KTN-PEG thiol-ene reaction [33].

### 3.2. Release of Unbound Keratins

**Figure 4:**
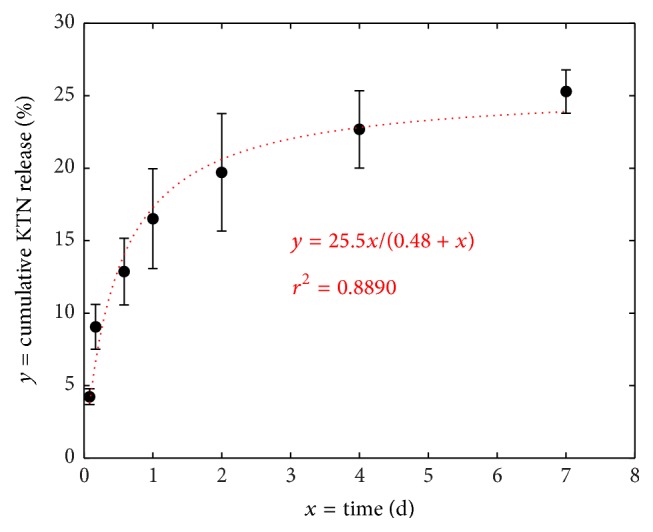
Rectangular hyperbolic release of uncrosslinked keratin (KTN) out of KTN-PEG scaffolds in PBS. The fitted equation predicted the maximum KTN release at 25.5%, half of which was released in just 0.48 days.

Despite statistical similarities in the *m*_absorbate_/*m*_scaffold_ ratio among KTN-PEG time groups, there was a downward plateauing trend over time (Figure 3), suggesting that some of the water-absorbing keratins were initially being released into the PBS medium. Total protein assay showed that uncrosslinked keratin proteins were indeed leaving the bulk of the scaffolds (Figure 4) in a rectangular hyperbolic fashion (*r* ^2^ = 0.8890) following Michaelis-Menten kinetics,

(4) $$\begin{array}{c} y=\frac{V_{max}\cdot x}{K_{M}+x}, \end{array}$$

where *x* is time (d), *y* is release concentration ratio, *V*_max_ is maximum release, and *K*_*M*_ is time at half *V*_max_. *V*_max_ was found to be 25.5%, implying that 74.5% of keratins were stably integrated into the KTN-PEG scaffold network. At 0.48 days or about 11.5 hours (*K*_*M*_), half of the unbound keratins were released in a static PBS environment at 37°C. Overall, washing and rinsing of the scaffolds in PBS on a shaker accelerated the removal of “free” or nonnetwork keratins.

### 3.3. Absorption and Then Release of Proteins in Neutral and Acidic pH

**Figure 5:**
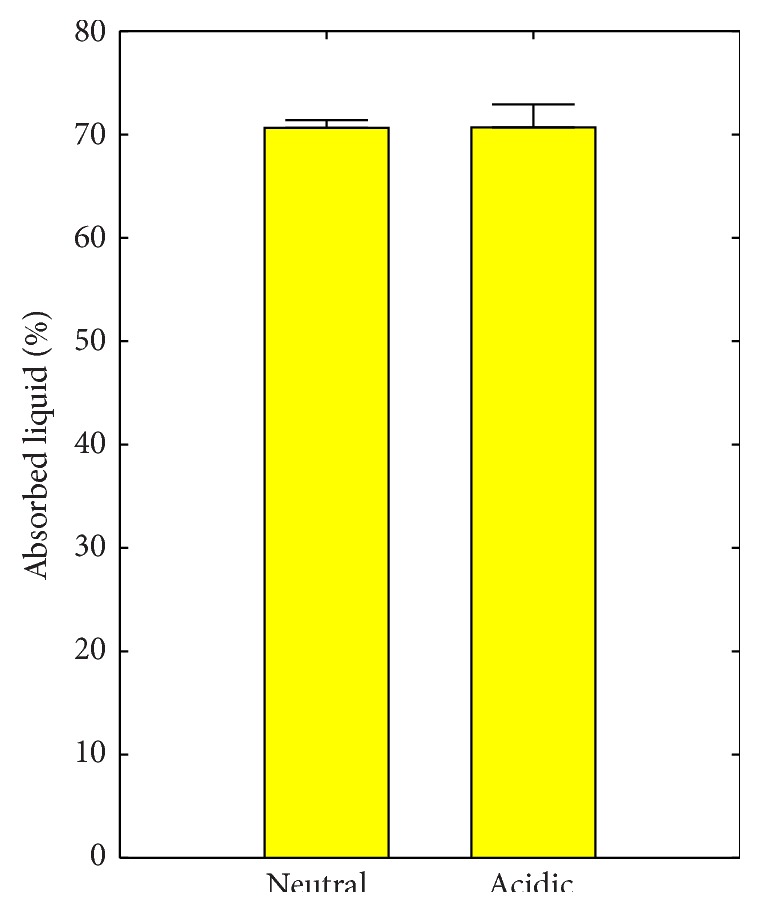
Liquid absorption at different pH levels: neutral (pH 7.4) and acidic (pH 4).

KTN-PEG scaffolds absorbed 70.7 ± 1.4% liquid (*V*_liquid_/(*V*_liquid_ + *V*_scaffold_)) containing 200 *μ*g/mL of test globular proteins (Alb, Hem, or Lys). This value can also be interpreted as the gel water content, determined to be less than reported 82% in Table 2. The discrepancy can be accounted for by the loss of uncrosslinked keratins, thereby slightly decreasing the capacity to hold water into the scaffold matrix. No significant difference (*p* = 0.9627) was observed between the liquid absorption behavior in neutral (pH 7.4) and in acidic (pH 4) PBS (Figure 5; 70.6 ± 0.7% versus 70.7 ± 2.2%, resp.).

**Figure 6:**
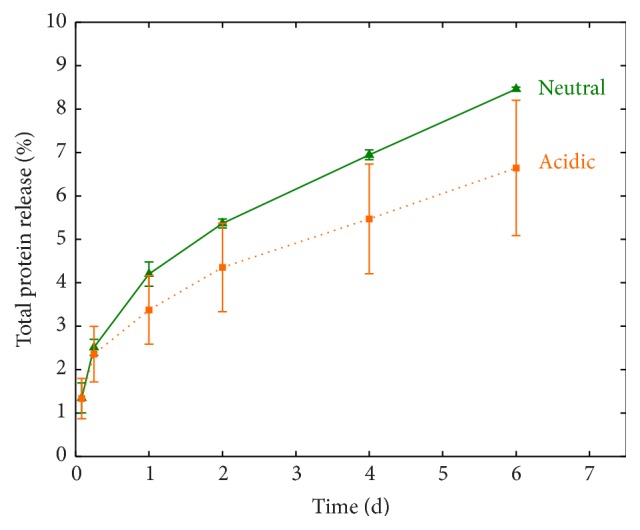
Release of crosslinked KTN (keratin degradation) in neutral and acidic PBS. Degradation in acidic pH 4 generated more variability among samples but at a slower trend relative to those in neutral pH 7.4.

Keratins covalently linked into the scaffold network degraded and released proteinaceous materials into the surrounding liquid medium at a relatively slow pace, 8.46 ± 0.05% in neutral PBS versus 6.64 ± 1.56% in acidic PBS for 6 days (Figure 6), although there was no significant difference between them (*p* = 0.1139). The keratin degradation profile was relatively slower with higher variability (standard deviation) at the pH of 4, suggesting that the scaffold has more stability in acidic pH. This was consistent with our previous findings in experiments of keratin gel degradation at varying pH [22], possibly because disulfide bonds were more protected during hydrolysis in acidic pH levels [34].

**Figure 7:**
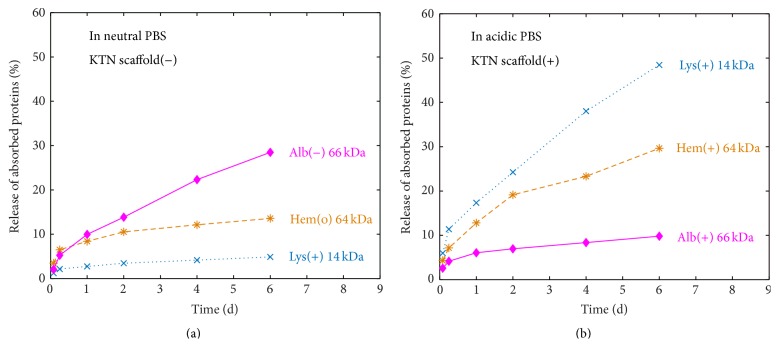
Release of absorbed proteins out of KTN-PEG scaffolds in (a) neutral (pH 7.4) and (b) acidic (pH 4) PBS media.

Proteins absorbed into the bulk gel matrix were released into the surrounding PBS through the processes of diffusion and osmosis: movement of mobile masses from higher to lower concentration gradients. The experimental setup allowed for high volume of PBS to scaffold ratio; hence the movement of molecules was not hindered by the saturation effect of the supernatant. In PBS pH 7.4, albumin was released the fastest, followed by hemoglobin and then lysozyme (Figure 7(a)). At this pH level, scaffolding keratin matrix proteins (with pI = 5.3) [22] are negatively charged (pI < pH; Table 1), dictating the overall charge density of the KTN-PEG sponge-like material. While albumin (pI ~ 5) is also negatively charged, hemoglobin (pI ~ 7) is almost neutral (though slightly negative) and lysozyme (pI ~ 11) is positively charged. The observed protein release rates can be explained by electrostatic interactions between charged species; that is, negative-negative repulsion drove albumin diffusion out the fastest. At day six, 28.5% of the original albumin load had been released into the medium. Conversely, the positively charged lysozyme electrostatically bound to the negative keratin backbone which consequently registered minimal release kinetics, at about the same rate as keratin degradation (4.9% for 6 days).

In an acidic environment, the amine groups of amino acids in proteins are more conducive to protonation (–NH_3_^+^), inducing net positive charges. Specifically at pH 4, the keratin matrix charge shifts to positive (Table 1). All loaded proteins, with pIs > 4, also now carry positive charges. Accordingly, repulsive forces dominate between the scaffold matrix and the absorbed globular proteins, thereby inducing even faster release of mobile proteins and the order dictated by size diffusion (the smaller the faster the release). The porous nature of the scaffold enables repulsion to force like-charged molecules out of the bulk material [35, 36]. Experimental results (Figure 7(b)) concurred with the projected kinetics wherein the smallest protein, lysozyme (14 kDa), had the highest rate of 48.4% in 6 days. Next in the order of release was hemoglobin (64 kDa) at 29.6% and finally albumin (66 kDa) at 9.8% in the 6-day period. Both lysozyme and hemoglobin were released at a higher rate in pH 4 than in physiological pH (Figure 7(b) versus Figure 7(a)). Albumin, on the other hand, had a lower rate of release in acidic medium, suggesting that the positive-positive repulsive force (^+^KTN and ^+^Alb) is relatively weaker than the negative-negative interaction (^−^KTN and ^−^Alb). Another possible explanation is that the low pH condition enabled the exposure of hydrophobic regions in both keratin [37] and albumin [38] resulting in hydrophobic attraction and slower albumin release.

Growth factors (GFs) are proteins secreted by cells targeting recipient cells via binding and interaction to cell-surface growth factor receptors leading to a variety of downstream effects [2–4] including local recruitment of repair stem cells for tissue engineering and regenerative medicine applications [39]. They can be held into the scaffold matrix through affinity-based methods [6] for sustained delivery and extension of efficacy [40]. Lysozyme can act as an inexpensive model for studying the kinetics of GF release since its size and isoelectric point (charge) properties are close to clinically relevant GFs [3] such as bone morphogenetic protein 2 (BMP-2; MW = 18 kDa (monomer); pI = 9) [41, 42] and brain-derived neurotrophic factor (BDNF; MW = 14 kDa (monomer); pI = 9) [43]. At neutral pH, lysozyme was sequestered tightly within the scaffold but was extensively released in an acidic condition (Figure 7) when the KTN charge flipped from negative to positive. GFs loaded into the KTN-PEG construct are thus anticipated to behave similarly to lysozyme. For evaluation, vascular endothelial growth factor C (VEGF-C; MW ~ 15 kDa (monomer); pI = 8.3) [26, 27] solution was utilized as an absorbent and its retention and activity were assessed indirectly on the survival of endothelial cells.

### 3.4. Interaction of Endothelial Cells on VEGF-C-Absorbed KTN-PEG Scaffold

**Figure 8:**
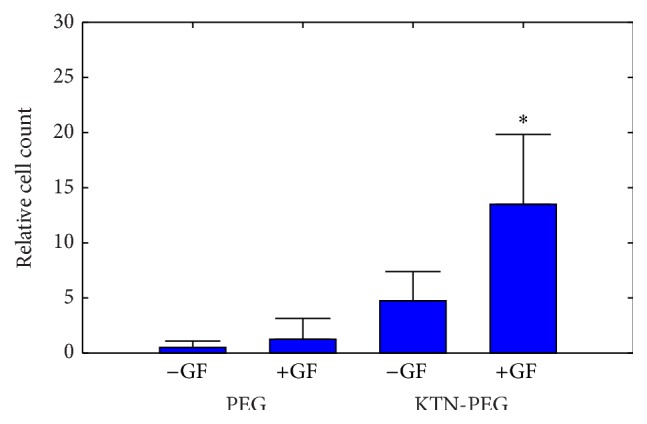
Endothelial cell culture on scaffolds, with (KTN-PEG) and without (PEG) keratins, in the presence (+GF) and absence (−GF) of absorbed VEGF-C. ^*∗*^ *p* < 0.05 compared to the other groups.

Fluorescence microscopy revealed no dead cells (no propidium iodide, red fluorescence staining) for any of the adherent cells. Endothelial cells (calcein AM for living cells, green fluorescence signals) cultured on KTN-PEG scaffold with electrostatically bound VEGF-C showed statistically more (*p* < 0.05) cell attachment and survival relative to the other three groups: KTN-PEG without VEGF-C, PEG with VEGF-C, and PEG without VEGF-C (Figure 8). The bar graph trend indicated that the presence of keratin as a network material generally increased cell growth, likely because of cell-interaction with the inherent cell-binding domains of keratins [44]. Addition of the growth factor VEGF-C further increased the number of cells on the scaffold surface suggesting that VEGF-C bound to the immobilized keratin network allowed cells to interact via their growth factor receptors facilitating attachment and promoted viability. Despite the outcome, the number of cells that anchored onto the substrate material was still relatively low. Application of cyclic sinusoidal pressure may induce endothelial cell proliferation with VEGF-C exposure [45]. Cultured lymphatic endothelial cells may respond better to the sequestered VEGF-C due to VEGF-C's importance in lymphangiogenesis or the formation of lymphatic vessels [46–48]. Future experiments may also require addition of surface-incorporated integrin-binding molecules such as laminin and its cell-binding peptides, RGD, YIGSR, and IKVAV, to improve the initial endothelial cell attachment [49].

## 4. Conclusions

Keratin (KTN) proteins in the recent years are gaining interest as appealing biocompatible biomaterial carriers of potent therapeutics which include growth factors. We have integrated and immobilized KTN with polyethylene glycol (PEG) diacrylate (PEGDA) through photoactivated linkage of KTN free thiols with PEGDA acrylate ends forming a stable KTN-PEG scaffold. KTN in the scaffold matrix enabled increased water uptake along with solubilized proteins. Its water-absorption capacity was found to be similar in physiological phosphate-buffered saline (PBS) pH 7.4 and in PBS pH 4. This network-bound KTN provided low hydrolytic degradation rate over the 7-day period with trend suggesting an even slower KTN release in acidic pH. Scaffold-absorbed proteins interacted with the KTN bulk following the First Law of Electrostatics: like charges repel and opposite charges attract. At pH 7.4, the negatively charged KTN tightly held onto proteins with basic isoelectric points (pIs) while preserving bioactivity. Lowering the pH to 4, below KTN's pI of 5.3, induced the fast release of a sequestered protein. The fabricated KTN-PEG construct can potentially be used as a slowly degradable sponge-like material for burst-release of high quantities of growth factors in acidic environment but also attract endogenous positively charged growth factors in neutral to basic pH states.

## Figures and Tables

**Table 1:** Charge-based protein interaction.

Role	Protein	pI	Medium condition			
			pH 7.4		pH 4	
			Net charge	KTN interaction	Net charge	KTN interaction
Scaffold	Keratin (KTN)	5.3	−	Repulsion	+	Repulsion
Drug load	Albumin	~5	−	Repulsion	+	Repulsion
Drug load	Hemoglobin	~7	0	None	+	Repulsion
Drug load	Lysozyme	~11	+	Attraction	+	Repulsion

**Table 2:** KTN-PEG scaffold properties.

	Lyophilized	Swollen (in PBS)	Unit
Density	1.09 ± 0.04	1.02 ± 0.15	g/mL
Water content	36 ± 2	82 ± 12	% (V/V)
[PEG]	59 ± 3	17 ± 3	% (m/V)
[KTN]	10 ± 1	3 ± 0	% (m/V)

**Table 3:** Scaffold network properties.

	*Q*	*E*_*c*_	*ν*_*e*_	*M*_*c*_
		(MPa)	(*μ*mol/mL)	(kDa)
PEG scaffold	2.6 ± 0.3	0.88 ± 0.35	257 ± 103	5.0 ± 2.4
KTN-PEG scaffold	3.0 ± 0.2	0.15 ± 0.01	42 ± 3	25.9 ± 1.7
